# Exploring the influence of vaping on the pharmacokinetic fate of inhaled therapeutics

**DOI:** 10.1007/s00204-025-04060-w

**Published:** 2025-04-27

**Authors:** Merna Adam, Madeline Bain, Toufic Ashraf, Jayden Dona, Borouj Al Zaben, Gina Shafik, Ramya Srikantharajah, Mangesh Pradeep Kulkarni, Kylie A. Williams, Gabriele De Rubis, Stewart Yeung, Brian Gregory George Oliver, Kamal Dua

**Affiliations:** 1 Discipline of Pharmacy, Graduate School of Health, University of Technology Sydney, Ultimo, NSW 2007 Australia; 2 Faculty of Health, Australian Research Consortium in Complementary and Integrative Medicine, University of Technology Sydney, Ultimo, NSW 2007 Australia; 3 Woolcock Institute of Medical Research, Macquarie University, Sydney, NSW 2113 Australia; 4 School of Life Sciences, University of Technology Sydney, Ultimo, NSW 2007 Australia

**Keywords:** Vaping, Smoking, Lung diseases, Inhaled drugs, Pharmacokinetics

## Abstract

The surge of electronic cigarette use in Australia, especially amongst the younger population, raises significant concerns about its impact on respiratory health. This review focuses on the detrimental effects of vaping on pulmonary function, delving into oxidative stress, ventilation–perfusion mismatching, as well as cellular damage. Our findings show that e-cigarette use adversely affects the pharmacokinetics of inhaled therapies, reducing efficacy through impaired drug distribution, clearance and absorption, as well as alterations in metabolism. These negative effects mirror the impacts of traditional cigarette smoking, posing a severe health risk not only to individuals who vape, but also to those with pre-existing respiratory conditions. Despite its perception as a safer alternative, its consequence on pulmonary health is becoming increasingly evident with issues such as nicotine addiction and emerging evidence that even short-term exposure to e-cigarette aerosols impairs lung function, potentially paving the way for chronic respiratory diseases. This underscores an urgent need for further research on its long-term implications, particularly for individuals relying on inhalation therapies, emphasising the need for informed public health strategies and guiding clinical practice to safeguard respiratory health in this rapidly evolving landscape.

## Introduction

The use of e-cigarettes has increased significantly over the past decade in Australia. Between 2019 and 2022–2023, the prevalence of vaping tripled, with 7% of the population using e-cigarettes compared to 2.5% in 2019. This increase was particularly significant among younger individuals, with nearly 50% of those aged 18–24 years and around 28% of those aged 14–17 years reporting they had tried vaping. Younger users were primarily driven by curiosity and the perception that e-cigarettes tasted better than traditional cigarettes, while older users often saw vaping as a tool to quit smoking (Welfare 2022–2023). The introduction of stricter legislation surrounding provision of vapes and vaping products in 2024 has corresponded with a decline in vaping rates of 28%, 46% and 36% for age groups 15–29, 30–59 and 15 + years, respectively (Australia 2025). However, the use of electronic cigarettes, particularly amongst younger individuals, is still identified as an issue with 66% of parents and 88% of teachers reporting concern surrounding these behaviours (Jenkinson et al. 2024).

Despite claims by manufacturers that e-cigarettes are a safer alternative to traditional tobacco-containing cigarettes, the World Health Organization advises individuals to remain cautious due to the lack of conclusive evidence on their long-term safety and efficacy as a smoking cessation tool (Marques et al. 2021). A recent study identified that e-cigarettes may be more likely to help individuals quit smoking in comparison to nicotine replacement therapy, with 18% utilising e-cigarettes to quit, and 9% using nicotine replacement therapy. However, many users reported increased throat and mouth irritability with e-cigarette use (Hajek et al. 2019). Additionally, adherence to treatment after 1 year of smoking cessation was significantly higher amongst participants using e-cigarettes (80%) compared to those using nicotine replacement therapy (9%). This difference may be due to e-cigarettes’ ability to reduce tobacco smoke withdrawal symptoms (Hajek et al. 2019). For patients with chronic obstructive pulmonary disease (COPD), switching to e-cigarettes has led to reduced cigarette consumption and fewer flare-ups, ultimately improving their physical activity levels (Marques et al. 2021). However, several studies have highlighted contradictory evidence regarding the efficacy and long-term use of e-cigarettes as a smoking cessation tool. These studies found insignificant correlations between e-cigarette use and higher rates of quitting smoking among traditional cigarette smokers (Patil et al. 2020). Furthermore, the use of e-cigarettes is associated with an increased risk of relapse in previous smokers, showing a 70% increased chance of smoking relapse (Gomajee et al. 2019). In addition, there has been an increased risk of smoking initiation in people who have previously vaped. Individuals who had any history of vaping had 4.9 times higher chance of smoking cigarettes as opposed to individuals who had no history of vaping. This was especially noted in younger adolescents aged 12–14 years, which was previously underestimated in other studies by 44% (Egger et al. 2024). Therefore, the long-term safety and efficacy of e-cigarettes remain uncertain; users may continue to vape after quitting traditional smoking and face risks of relapse, or engage in dual use of both vaping and traditional smoking. It is shown that e-cigarettes are often used for dual use more frequently than traditional nicotine replacement therapies. Dual use refers to using both e-cigarettes and traditional cigarettes simultaneously. This can discourage complete smoking cessation, as individuals continue smoking traditional cigarettes alongside e-cigarettes instead of complete tobacco cessation. As such, further research remains critical to fully understand the long-term safety and efficacy of vaping (Feeney et al. 2022).

## Key elements of e-cigarettes

The use of e-cigarettes, or alternatively, vaping, is the action of inhaling a resulting vapour, which is initiated through the activation of an atomiser, heating the liquid within a reservoir. E-cigarettes are battery-powered devices that, within the reservoir liquid, mostly contain nicotine, flavouring, and other chemicals such as acetone, acrolein and 2-chlorophenol. As of 2022, there are 460 different English e-cigarette brands on the market (Abuse 2020).

The primary structural components of e-cigarettes are the mouthpiece, cartridge, atomiser and battery (Kaur et al. 2018). The liquid in the cartridge consists of vegetable glycerine and propylene glycol with a small percentage of nicotine and flavourings (Famele et al. 2015). The concentration of nicotine is 16–24 mg/mL in e-cigarettes, which typically contain 2 mL of e-liquid that is much greater than that of traditional cigarettes, with each containing 10–14 mg of nicotine (Traboulsi et al. 2020). The atomisation of vegetable glycerine, propylene glycol and flavourings in e-liquids produces carbonyl compounds such as formaldehyde, acetaldehyde and acrolein (Famele et al. 2015). Furthermore, the heating of the atomiser can release heavy metals such as chromium and nickel (Kaur et al. 2018). The extraction process of nicotine from tobacco may cause impurities such as N-nitrosamines and volatile organic compounds such as benzene to be incorporated into the e-liquid (Famele et al. 2015). Inhalation of these by-products from the e-cigarette liquid, atomiser and extraction process may lead to severe respiratory diseases such as lung cancer due to their toxicity (Famele et al. 2015; Kaur et al. 2018). The specific effects of each by-product can be seen in Table 1.

**Table 1:** Chemical by-products of e-cigarettes and their associated pulmonary impact

E-cigarette chemical by-product	Pulmonary effect	References
Formaldehyde	Respiratory irritation, coughing, wheezing, pneumonia, chronic bronchitis, lung cancer	Kaur et al. (2018)
Acetaldehyde	Coughing, pulmonary oedema, lung cancer	Kaur et al. (2018), Traboulsi et al. (2020)
Acrolein	Respiratory irritant, possible carcinogen	Kaur et al. (2018)
N-nitrosamines	Lung cancer	Kaur et al. (2018)
Volatile organic compounds (benzene)	Lung cancer	Traboulsi et al. (2020)
Heavy metals (chromium, nickel)	Lung cancer, chronic bronchitis	Traboulsi et al. (2020)

## Lung function and features in non-vaping airways

Lung function is closely related to the structure and integrity of the airways and its efficiency in gas exchange. Some important mechanisms that maintain the integrity of the lung are its cellular components, epithelial lining and structure. Some cellular components encompass a variety of phagocytes, such as macrophages and neutrophils, which comprise the innate immune system. These phagocytes are crucial in providing defence against inflammation, damaged tissue, pathogens and infections and target harmful cells in the body (Bals and Hiemstra 2004). The epithelial lining functions as a protective barrier and is the first point of contact for inhaled substances such as pollution, smoke and allergens and is composed of mucus, cilia, antimicrobial peptides and cell junctions (Ganesan et al. 2013). One of its primary defence mechanisms is the mucociliary component, where mucus collects harmful particles, and cilia transports the mucus out of the lungs in unison. Mucus hydration and ciliary beating are essential for this process to be effective, and in healthy individuals, mucus secretion occurs regularly and is balanced, maintaining lung integrity (Bustamante-Marin and Ostrowski 2017).

Lung integrity is also maintained by the structure of the lungs, where it forms a tracheobronchial tree, spanning from the trachea and down to gaseous exchange in the alveoli (Verma et al. 2015). The efficiency and integrity of the lung structure, phagocytes and mucociliary clearance are crucial, as an impairment in the function and structure can lead to respiratory conditions such as asthma, COPD and cystic fibrosis (Munkholm and Mortensen 2014).

## Lung inflammation in the absence of e-cigarette smoke

The process of inflammation is vital in the maintenance of respiratory health and is mainly facilitated by the recruitment of macrophages and neutrophils in response to pathogens and tissue injury. Macrophages are flexible immune cells that are derived from monocytes that divide into two polarisation states—pro-inflammatory (M1) and anti-inflammatory (M2)—based on the stimuli encountered (Cheng et al. 2021). Upon exposure to pro-inflammatory signals such as interleukin-1 (IL-1), interleukin-6 (IL-6) and tumour necrosis factor-alpha (TNF-α) cytokines, M1 macrophages activate further pro-inflammatory responses such as additional cytokines, neutrophils and T cells that produce a heightened inflammatory response at the site of infection or injury. M2 macrophages are often stimulated by interleukin-10 (IL-10) and transforming growth factor-beta (TGF-β) cytokines and are responsible for anti-inflammatory tissue repair and regeneration (Cheng et al. 2021). Neutrophils comprise mostly white blood cells and are activated by chemical stimulus from damaged tissue and invading organisms. They are responsible for phagocytosis, degranulation and the generation of reactive oxygen species to eliminate and contain pathogens while avoiding damage to surrounding cells (Jasper et al. 2019).

Overexpression of neutrophils and macrophages may result in cell and lung tissue damage as well as chronic inflammation, as shown in traditional cigarette smoking and, if unresolved, may lead to fibrosis, impaired lung function and structural changes in the airway (Cheng et al. 2021). On the other hand, inadequate function of such immune cells compromises the lungs’ ability to respond to harmful substances, increasing infection and inflammation susceptibility. As a result, maintaining optimal respiratory defence requires a balance in immune cell expression.

## Different types of inhaled drug delivery

Inhalers are medical devices frequently used to prevent and relieve symptoms of respiratory conditions such as asthma and COPD (Sorino et al. 2020). There are four main types of inhalers, and each is designed to work differently in achieving the same goal. Pressurised metered-dose inhalers (pMDI) are most frequently utilised, where they deliver a precise medication dose in a pressurised and aerosolised form, and individuals must press the canister to release the medication dose (Usmani 2019). Dry powder inhalers (DPI), unlike others, are composed of microparticles in a powder form and the medication is activated upon quick and deep inhalation. Soft mist inhalers (SMI) support the transformation of aqueous medicines into a fine mist form, allowing for ease of use. A nebuliser is a machine that requires the patient to use a mask or a mouthpiece to help deliver the medication through a constant fine mist directly to the lungs, where it can deliver larger doses of medication compared to other inhalers, making it ideal for acute exacerbations (Usmani 2019).

## How do inhaled therapies work?

Inhaled drug delivery systems have proven to be an optimal method for drug absorption in the treatment of pulmonary conditions (Ibrahim et al. 2015). The drug is administered directly to the site of action, allowing a quick onset of therapeutic effect. For this to be successful, the delivery system and drug must possess specific characteristics to overcome the lungs’ natural defence barriers and allow for adequate absorption (ElKasabgy et al. 2020). Whilst the specific mechanism of action (MOA) of the various types of inhalers differs, they share a common goal in aiming to deliver drug molecules to the target tissues and maintaining binding for an adequate time to elicit an effect. It has been determined that the low solubility of a delivered drug, as well as the delivery of a drug in its neutral form, can increase the retention time at the target site, allowing for a longer duration of action (Patton and Byron 2007).

Inhaled drugs and their delivery devices must be formulated to overcome the lungs’ inherent barriers. As previously detailed, the respiratory tract consists of a series of bronchiole bifurcations that progressively decrease in size until reaching the alveoli. The aerodynamic properties of drugs determine the location of drug deposition. Deposition of larger particles (> 5 μm) tends to occur in the mouth and upper respiratory tract, whilst drugs that are minute (< 0.5 μm) are instead cleared via exhalation (Borghardt et al. 2018). Therefore, delivery devices are designed to release particles of drug contents sized between 1 and 5 μm, allowing for deposition into the target sites of the bronchiole tissue (Borghardt et al. 2018). Mucociliary clearance is a natural mechanism of the lungs that aims to remove foreign particles by trapping and expelling them in the mucus layer (Guo et al. 2021). Large, coarse particles are likely to be cleared by this clearance mechanism, which further reinforces the importance of a drug particle size range of 1–5 μm (ElKasabgy et al. 2020). To minimise systemic absorption, such administered drug particles must have increased airway selectivity and low oral bioavailability (Borghardt et al. 2018). This ensures these particles reach the target bronchiole tissues and reduce the amount of drug that reaches the systemic system, lowering the risk of adverse events (Guo et al. 2021).

The current primary use of inhaled drugs is to exhibit a local effect on the lung tissue by binding to receptors on the bronchial smooth muscle in cases of bronchoconstriction. However, upon depositing in the lungs, these drugs are at risk of undergoing clearance, degradation via metabolism and systemic absorption (Labiris and Dolovich 2003). The duration of the therapeutic effect is dictated by the residency time of the drug within the lung/receptor site, which can be manipulated by changing the chemical properties of the drugs delivered via inhaled delivery devices (Labiris and Dolovich 2003). For example, administering ciprofloxacin via DPI in its neutral form slows the dissolution rate, increasing the residency time at the target site (McShane et al. 2018).

Whilst this process is evidently complicated, pulmonary drug delivery provides many benefits for the patient. It allows for the drug to be delivered directly to the target site, as well as for the avoidance of first-pass metabolism, ultimately requiring lower drug doses (Anderson et al. 2022). Furthermore, as less drug reaches the systemic circulation in comparison to other routes of administration, there is a lower incidence of systemic adverse effects (Rau 2005).

The absorption of inhaled drugs and the efficacy of pulmonary drug delivery systems depend largely on the physiology and characteristics of the lung tissues. Exposure of lung tissue to e-cigarette vapour has been linked to fluctuations in tissue composition as well as structural damage (Ghosh et al. 2018; Park et al. 2022). Oxidative stress, cell death as well as the impairment of mucociliary clearance have been correlated with e-cigarette use (Auschwitz et al. 2023; Chung et al. 2019; Gaurav 2019; Kwon et al. 2019; Lerner et al. 2015). These physical changes may have the potential to interfere with regular lung mechanisms and alter the lungs’ functionality with other foreign substances, such as inhaled drug particles. Throughout this manuscript, we aim to determine the effect of electronic cigarettes on the absorption and pharmacokinetics of inhaled drugs. It is hypothesised that e-cigarette use will induce pulmonary inflammation that will impact the efficacy and pharmacokinetics of inhaled medicines, mirroring traditional cigarette smoking.

## Effect of e-cigarette exposure on the airway epithelium

In this section, we cover the harmful effects of e-cigarettes on airway epithelium. In particular, this manuscript discusses oxidative stress, inflammation, vascularisation and perfusion.

Vaping exerts a detrimental effect on the airway epithelium, leading to significant cellular damage in the lungs and disruption of mucociliary clearance, both of which are important in the normal functioning and health of the lungs and airways. Lung epithelial tissues act as the first line of defence against foreign pathogens, chemicals and other potentially harmful substances, all of which enter the respiratory tract with each breath (Gaurav 2019). E-cigarettes contain various toxicants, such as aldehydes and heavy metal particles, which can exert direct cellular damage and increase oxidative stress within airway epithelial cells (Auschwitz et al. 2023). Furthermore, alteration of mucociliary clearance has been shown to occur because of exposure to e-cigarette vapour in a nicotine-dependent manner (Chung et al. 2019). Ciliated cells in the lungs play an important role in mucociliary clearance, which is an innate defence mechanism of the lungs to remove inhaled pathogens and particles (Bustamante-Marin and Ostrowski 2017). Alteration of mucociliary clearance is attributed to the fact that nicotine exposure increases mucus viscosity and secretion. This was indicated by a greater fluorescence recovery after photobleaching (FRAP) half-life in a 2019 study (Chung et al. 2019). This increase in mucus viscosity hinders the transport of mucus by cilia, causing a buildup of mucus in the airways, leading to breathing problems and an increase in susceptibility to infections (Auschwitz et al. 2023).

## E-cigarette use as a cause of oxidative stress

Oxidative stress arises from an imbalance between the accumulation of reactive oxygen species (ROS) in cells and tissues, and the body’s ability to neutralise or detoxify these reactive products. These free radicals predominate and can interfere with signalling pathways, ultimately resulting in cell and tissue damage (Pizzino et al. 2017). Developing research indicates that usage of electronic cigarettes can cause oxidative stress in human bronchial epithelial cells as well as in mouse lungs (Bonner et al. 2021). One study demonstrated that the exposure of wild-type C57BL/6J mice to electronic cigarette aerosols resulted in decreased total and oxidised levels of lung glutathione (Lerner et al. 2015). Glutathione is considered a fundamental antioxidant and exhibits its effect through the mechanism of reversible glutathionylation of the active thiols in proteins (Kwon et al. 2019). Alterations in its presence or activity within the lungs have been linked to increased oxidant-induced inflammation (Rahman and MacNee 1999). Furthermore, it has been found that bronchoalveolar levels of glutathione can be lower in patients with lung diseases such as COPD (Barnes 2020).

This same study also found that exposing human airway epithelial cells (H292 cells) to electronic cigarette aerosols resulted in a dose-dependent increase in pro-inflammatory cytokines IL-8 and IL-6 (Lerner et al. 2015). These results are supported by the findings of an in vitro study which exposed human lung adenocarcinoma epithelial cells (A549) to increasing levels of electronic cigarette vapour extract concentrations: 100% vapour extract with one in ten serial dilutions. The study found that electronic cigarette exposure increased ROS formation whilst decreasing glutathione-associated metabolites (Assiri et al. 2024). The nuclear factor kappa B (NF-κB) pathway plays a significant role in controlling ROS levels within the body through the expression of antioxidant proteins (Liu et al. 2017). Mutations in this pathway have been linked to the pathogenesis of inflammatory diseases such as rheumatoid arthritis and COPD (Pai and Thomas 2008). The effects of electronic cigarettes on this pathway were tested in an in vivo study using transgenic NF-κB-luc mice. The results revealed that when these mice were exposed to e-cigarette aerosol, this pathway was affected, inducing inflammation and oxidative stress (Ma et al. 2020). Ultimately, there are multiple studies highlighting the relationship between the use of electronic cigarettes and the promotion of ROS, which raises concern surrounding the impact this damage may have on individual health. Aldehydes such as formaldehyde generate reactive oxygen species (ROS) through their heating and vaporisation and cause inflammation in the lungs. Elevation of ROS levels results in oxidative stress in airway epithelial cells which activates an antioxidant defence mechanism known as the Keap1–Nrf2 pathway to counteract the harmful effects of oxidative stress (Schieber and Chandel 2014). Nuclear factor erythroid 2-related factor (Nrf2) is a transcription factor that is kept in the cytoplasm in an inactive form by its inhibitor Keap1. In the presence of ROS, the binding of Nrf2 and Keap1 is compromised, and Nrf2 enters the nucleus initiating antioxidant transcription. However, despite its protective role, the constant activation of the Nfr2 pathway in response to e-cigarette use, particularly the flavouring agents found in e-cigarettes, can exhaust this protective pathway, leading to eventual cellular death in the epithelium (Ma et al. 2020; Schieber and Chandel 2014).

## Changes in airway vascularisation and perfusion

Gas exchange takes place in the lungs between the air in the alveoli and the blood in the pulmonary capillaries. For gas exchange to be effective, the alveoli require proper ventilation and perfusion. Ventilation (V) refers to the movement of air into and out of the alveoli, while perfusion (Q) relates to the blood flow to the alveolar capillaries (Hopkins 2020). Exposure to e-cigarette smoke leads to alterations and mismatching in these essential mechanisms.

Such impact was examined in rats, where significant emphysematous changes and enlargement of the alveolar airspace were caused by exposure to e-cigarettes and nicotine, while concomitantly leading to a marked decrease in capillary density. This effect mirrored traditional cigarette smoking, whereby an alteration and mismatching in airway vascularisation and perfusion is present (Reinikovaite et al. 2018). Another study revealed that young and healthy e-cigarette users exhibit significant ventilation–perfusion mismatching, which worsens after a single vaping session. Despite normal spirometry and being asymptomatic, early signs of pulmonary vascular dysfunction were exhibited. There was an acute increase in pulmonary blood flow and heart rate, and a chronic response indicated increased perfusion heterogeneity (*P* = 0.006), suggesting that vaping may alter the extent of the pulmonary circulation response, akin to the characteristics of COPD (Puliyakote et al. 2020). These detrimental changes in pulmonary function mirror the impacts of traditional cigarette smoking, casting debate on its notion of it being a healthier alternative.

One consequence of vaping-induced ventilation–perfusion mismatch is an increased alveolar–arterial oxygen difference (AaDO2) which means oxygen is not transferring effectively and can result in hypoxemia (low blood oxygen levels) (Hopkins 2020). Some regions of the lungs may not receive sufficient air or blood flow which reduces the overall oxygenation capacity. Notably, changes in blood flow within the lungs may occur before any visible airway abnormalities. This suggests that vaping-induced vascular changes could lead to long-term declines in lung function even before noticeable symptoms appear (Puliyakote et al. 2020). Additionally, vaping may also disrupt the regulation of blood flow in the lungs, specifically the hypoxic pulmonary vasoconstriction response. Normally, this process constricts blood vessels in regions with low ventilation and redirects blood flow to well-ventilated regions to optimise gas exchange. However, vaping may disrupt this process, making ventilation–perfusion mismatch worse and increasing the risk of pulmonary conditions like COPD (Puliyakote et al. 2020).

Overall, disruptions in lung function from vaping can lead to early vascular damage that may not be detected by standard spirometry tests. While immediate effects may not be apparent, long-term exposure to e-cigarette aerosols can gradually impair oxygenation capacity and increase the risk of respiratory disease (Puliyakote et al. 2020).

## Acute impact of e-cigarette use on lung function

E-cigarette use has been shown to have significant consequences on the human respiratory system. The acute effects of vaping are relatively well understood and offer an immediate insight into its detrimental impact. Clinical symptoms were reported just 10 min after use, in a 2017 study*,* where most healthy smokers or those without any known respiratory issues, reported a dry mouth (79%) and sore throat (61%) after inhaling the nicotine-containing e-cigarette (Palamidas et al. 2017). Likewise, an acute cough was reported by 69% of smokers with COPD and 55% of people with asthma. Heart palpitations were reported by 46% of those with asthma, 19% of those with COPD and 36% of healthy smokers (Palamidas et al. 2017). Furthermore, inhalation of nicotine-containing e-cigarette vapours was shown to produce sudden effects on vital signs and lung function. A significant increase in airway resistance was detected in asthmatic (*p* = 0.034) and healthy smokers (*p* = 0.004). A statistically significant decrease in oxygen saturation (SpO_2_) was also recorded following e-cigarette use in healthy smokers (*p* < 0.001) and COPD smokers (*p* < 0.05) (Palamidas et al. 2017). At a molecular level, exposure of the bronchial epithelial cells to e-cigarette vapours leads to greater secretion of IL-8 and IL-6 expression in human lung fibroblasts, all of which indicate enhanced inflammatory response in the lungs. Other elevated inflammatory markers include tumour necrosis factor-alpha (TNF-α), transforming growth factor-beta (TGF-β), C–X–C motif chemokine ligand 8 (CXCL8), monocyte chemoattractant protein-1 (MCP-1) and matrix metalloproteinase-9 (MMP-9) (Masso-Silva et al. 2021).

## Long-term impact of e-cigarette use on lung function

The link between the use of chronic e-cigarettes and its negative impact on lung health has consistently been demonstrated throughout varying literature as a topic of concern. Essential proteomic and metabolic pathways demonstrate compromise in respiratory function when linked with vaping (Ghosh et al. 2018). In a 2017 study, long-term use of e-cigarettes triggered an increase in oxidative stress and inflammatory responses, fundamentally leading to impaired immune response. In addition, findings in other papers indicated an alteration in specific proteins, such as those related to the NF-kB pathway. These proteins play a role in cellular repair and antioxidant defence, leading to mitigated lung resilience, thus leading to severe respiratory conditions such as asthma, pulmonary fibrosis and COPD (Park et al. 2022). Similarly, it was found that prolonged exposure to e-cigarette vapour disrupts crucial metabolic pathways responsible for cellular repair and energy production. Evidence from these findings demonstrates that the disruption of these processes leads to the lung’s inability to maintain homeostasis and recover from injury, resulting in a reduction in mucociliary clearance. A reduction in mucociliary clearance poses a serious risk in the development of respiratory infections and potentially exacerbates chronic lung conditions (Assiri et al. 2024). In addition, essential proteins such as proteins 53 (P53) and heat shock proteins (HSP70) that play crucial roles in cellular repair and metabolic pathways have notably been downregulated when exposed to e-cigarette vapour. Furthermore, e-cigarette usage exposes individuals to harmful substances such as vitamin E acetate, which is an irritant to the lungs. Exposure to these, alongside other substances of this nature, has been linked to a severe lung condition: e-cigarette use-associated lung injury (EVALI) (Ghosh et al. 2018). Chronic exposure to these substances may lead to lifelong, lasting respiratory damage, such as increased susceptibility to laryngeal mucosa hyperplasia and metaplasia. This is due to the intense inflammatory and oxidative stress that comes with vaping. The overarching theme in these studies indicates a higher risk of developing chronic respiratory issues and lung diseases such as bronchitis, emphysema and lung cancer (Assiri et al. 2024; Ghosh et al. 2018).

## Effect of pulmonary damage on the pharmacokinetics of inhaled therapeutics

In this section, we review how damaged respiratory tissue and disrupted pulmonary mechanisms affect the pharmacokinetics of inhaled drugs, notably, discussing the effects upon absorption, distribution, metabolism and elimination of prevalent medications such as beta agonists and inhaled corticosteroids.

## Reduction in inhaled drug absorption

The effects of vaping on the lungs can impact the absorption of inhaled medications in individuals with respiratory conditions such as asthma, bronchiectasis and COPD. Research shows that brief exposure to e-cigarette vapour may lead to immediate changes in lung function, increasing inflammation and airway resistance (Kotoulas et al. 2020). Research utilising impulse oscillometry found that the increase in inflammation and resistance may occur within 5 min of vaping (Vardavas et al. 2012). Longer exposure increases these effects by raising exhaled nitric oxide (FeNO) levels and reducing vital capacity (VC) (Tsai et al. 2020). This suggests that vaping triggers airway inflammation and reduces lung function, both of which are essential for effective drug absorption in the lungs.

The inflammation and bronchoconstriction caused by vaping disrupt the normal pulmonary airflow, an essential mechanism for effective drug distribution and delivery (Traboulsi et al. 2020). In healthy lungs, inhaled medications are distributed evenly across the central and peripheral regions. However, in vaping-affected lungs, the increased airway resistance causes turbulent airflow. This results in most of the drug disposition occurring in the central regions of the lungs instead of reaching the peripheral areas where they are most required in achieving the most therapeutic benefit. This is particularly concerning for patients with asthma and COPD as their already compromised lung function is further weakened by vaping, potentially reducing the effectiveness of their inhaled treatments (Labiris and Dolovich 2003). Moreover, vaping may lead to eosinophilic inflammation in asthmatic patients by increasing the levels of Th2 cytokines, in particular, IL-4 and IL-13. This further reduces the lung’s ability to absorb inhaled drugs (Kotoulas et al. 2020). In addition, irritants and chemicals in e-cigarettes, including flavouring agents, also contribute to bronchospasm and mucosal swelling, further causing a decrease in the level of absorption of inhaled therapies (Tsai et al. 2020). These effects are similar to traditional tobacco smoking, where increased mucus production and airway thickening impair lung function (Darabseh et al. 2021). As vaping becomes increasingly popular, individuals with chronic respiratory conditions may experience worsening respiratory symptoms due to the decreased efficacy of their inhaled medications caused by a reduction in drug absorption in the lungs. In summary, early studies suggest that vaping impacts the absorption of inhaled therapies by causing an increase in airway resistance and significant inflammation.

## Reduced inhaled drug distribution

The components of electronic cigarettes may contribute to lung cancer, which can increase the expression of ATP-binding cassette (ABC) transporters. This, in turn, increases the cellular efflux of inhaled drugs, leading to reduced drug concentration within cells. The main ABC transporters in lung tissue are P-glycoprotein (P-gp), multi-drug resistance protein 1 (MRP1) and breast cancer resistance protein (BCRP) (Zwoliński et al. 2024). Inhaled therapies that require P-gp substrates include beclomethasone, budesonide, ciclesonide, mometasone, fluticasone furoate, olodaterol, vilanterol and umeclidinium. BCRP substrates are beclomethasone, budesonide, ciclesonide and mometasone, while MRP1 substrates include budesonide, formoterol and ipratropium (Nickel et al. 2016). A study found that tissue retention of most inhaled drugs was lower in lung epithelial cells of cancerous rats in comparison to Caco-2 cells, which consist of inhibited ABC transporters that mimic human cells (Eriksson et al. 2018; Fredlund et al. 2017). Similarly, another study in rats demonstrated that inhibition of P-gp, MRP1 and BCRP caused a direct retention of radiolabelled substrates in epithelial cells, increasing substrate exposure (Mairinger et al. 2022). Furthermore, electronic cigarettes increase the expression of transforming growth factor (TGF)-β, leading to a promotion of epithelial to mesenchymal transition (EMT), potentially leading to tumour growth (Zwoliński et al. 2024). EMT induced by e-cigarette smoke reduces E-cadherin expression, therefore increasing the permeability of drugs through the epithelial tight junctions in the alveoli, interrupting the lung’s barrier (Ahmad et al. 2018; Haghi et al. 2014). The presence of tumours may also impede the flow of inhaled drugs into the lower airways, as most drug particles tend to deposit in the upper airways due to obstruction by tumour growth (Man et al. 2023).

A decrease in forced expiratory volume in one second (FEV1) also highlights the impact of vaping on drug absorption. FEV1 reflects the amount of air exhaled in one second and is a key marker of airway obstruction, where a reduction in FEV1 is a sign of decreased respiratory function. Short-term vaping has been shown to reduce FEV1, despite the use of nicotine-free devices. This may limit the distribution of inhaled drugs, particularly to the peripheral regions of the lungs. Although bronchodilators can improve FEV1 and enhance drug delivery, the inflammation and obstruction caused by vaping reduces these benefits, leading to poorer treatment outcomes (Labiris and Dolovich 2003). As a result, patients who rely on inhaled therapies may find their treatment less effective, as the vaping-induced changes in the lungs prevent medications from reaching their intended target areas.

## Altered inhaled drug metabolism

In pulmonary drug delivery, medicines are inhaled through the lungs and enter the bloodstream via the alveolar epithelium. Drug administration into the respiratory tract allows for maximum drug absorption due to the lung’s large surface area, rapid uptake of the drug by the alveoli, and a rich blood supply contributing to high bioavailability (Vazda et al. 2021). However, the lungs also contain a source of xenobiotic metabolising enzymes, which can influence the pharmacokinetics and overall metabolism of inhaled drugs (Enlo-Scott et al. 2021). The major function of these enzymes in the lungs is to metabolise, detoxify and eliminate exogenous agents, such as environmental pollutants and drugs that enter the lungs, which may cause potential harm to lung tissue and function. Examples of major enzymes include cytochromes (CYP) P450, carboxyl esterase (CES), and flavin-dependent monooxygenase (FMO) (Enlo-Scott et al. 2021). Upon inhalation of an e-cigarette, the vapour containing nicotine and flavouring agents enters the airways, altering the activity of such xenobiotic metabolising enzymes by both upregulation and inhibition, causing both an increase and decrease in drug metabolism. A decrease in drug metabolism is shown in vapes containing flavouring agents such as benzaldehyde and cinnamaldehyde that are potent inhibitors of the CYP2A6 enzyme, which is primarily responsible for the metabolism of nicotine, leading to an increase in plasma concentration levels of nicotine (Winters et al. 2023). Although further research is required, this finding indicates that nicotine-containing vapes, when inhaled together with these flavouring agents, could lead to an increase in systemic nicotine concentration, leading to a variety of adverse health events such as an increase in blood pressure, a sharp increase in adrenaline levels and a rapid heart rate, all increasing the risk of experiencing a heart attack. It has also been established that the liquid solution of e-cigarettes containing propylene glycol (PG) and vegetable glycerine (VG), which assist in producing the e-cigarette vapor, have been associated with impairing the growth and viability of the epithelial cells lining the airways (Woodall et al. 2020). This has been demonstrated through the effects of PG and VG on glucose transport, specifically their inhibition on cellular glucose uptake in proliferating airway cells, reducing cell surface area and leading to cell shrinkage. The alveoli, which are lined with epithelial cells, contain esterases, such as carboxylesterase 1 (CES1), which play a role in drug metabolism through prodrug activation or detoxification. Therefore, as the epithelial cells are being damaged through the inhalation of these e-cigarette constituents, drug metabolism will be altered, and there will be a reduced conversion of prodrugs into their active forms. For example, beclomethasone dipropionate (BDP), an inhaled glucocorticoid indicated for the maintenance treatment of asthma, relies on its conversion to its major active metabolite beclomethasone-17-monopropionate (17-BMP) to achieve its therapeutic anti-inflammatory effects (Qian et al. 2022). However, as these esterases responsible for the conversion of BDP to 17-BMP are damaged, less active drug will be available to the lungs, leading to reduced suppression of airway inflammation and worsening of asthma symptoms.

## Reduction in inhaled drug clearance

Inhaled medication clearance depends on the integrity of the lung’s structure and function. Vaping alters lung tissues both structurally and chemically, which significantly impacts drug clearance from the lungs and body (Taburet et al. 1990). Inhaled drugs can be eliminated from the lungs and primarily absorbed into the bloodstream, as they generally bypass the first metabolism. The primary method of elimination in the lung occurs through mucociliary clearance, where drug particles are captured by the mucus and moved by cilia towards the throat for swallowing, meaning that even after absorption into the bloodstream, drug particles can be cleared from the lungs for potential reabsorption and metabolism. Impaired mucociliary clearance, therefore a buildup in mucus, can cause drugs to accumulate in the lungs for an extended period, increasing drug toxicity and the risk of side effects. Impaired mucociliary clearance, which can occur as a consequence of vaping, can lead to mucus accumulation in damaged lung tissue, causing drugs, like bronchodilators or inhaled corticosteroids (ICS), to remain in the airways longer, slowing clearance rate, increasing the local concentration and side effects and ultimately altering its efficacy (Winters et al. 2023). Elimination of drugs in damaged lung tissue can be affected by impaired mucociliary clearance, which is caused by vaping and leads to the accumulation of drugs in the airways. This increases local drug concentration, adverse events and drug efficacy.

Impaired mucociliary clearance directly affects treatment outcomes as shown in patients with COPD. Accumulation of mucus in the lungs is associated with ineffective pathogen removal and increased airway obstruction, creating an environment where infections can persist and trigger inflammatory response (Thomas et al. 2021). These lead to worsening airway resistance, insufficient airflow, shortness of breath, an increased lung effort to breathe as well as exercise intolerance (Bhowmik et al. 2009). These are key markers indicative of COPD exacerbations, impacting quality of life and directly affecting treatment outcomes.

Reduced mucociliary clearance in the lungs due to e-cigarettes will increase the exposure of inhaled corticosteroids and increase the risk of systemic side effects (Edsbäcker et al. 2008). Examples of systemic side effects of inhaled corticosteroids relevant to the lungs are viral respiratory infections and tuberculosis (Pandya et al. 2014).

When an 18-year-old female with a history of asthma started vaping cannabinoid oil twice weekly, her health condition deteriorated. Vaping reduces surfactant production and function, mucociliary function and the CFTR channel, leading to lung injury. This resulted in symptoms such as night sweats, fatigue and cough lasting for 1 week. A chest CT scan showed a large cavity in the left upper lobe, numerous calcified nodules on both lungs and swelling of the lymph nodes between the lungs. Sampling of the lung tissue demonstrated a fungal infection, which was treated with posaconazole, and a bacterial infection, which was treated with rifabutin, ethambutol, azithromycin, moxifloxacin and amikacin. Most symptoms resolved within 1 month (Chen et al. 2021).

**Fig. 1:**
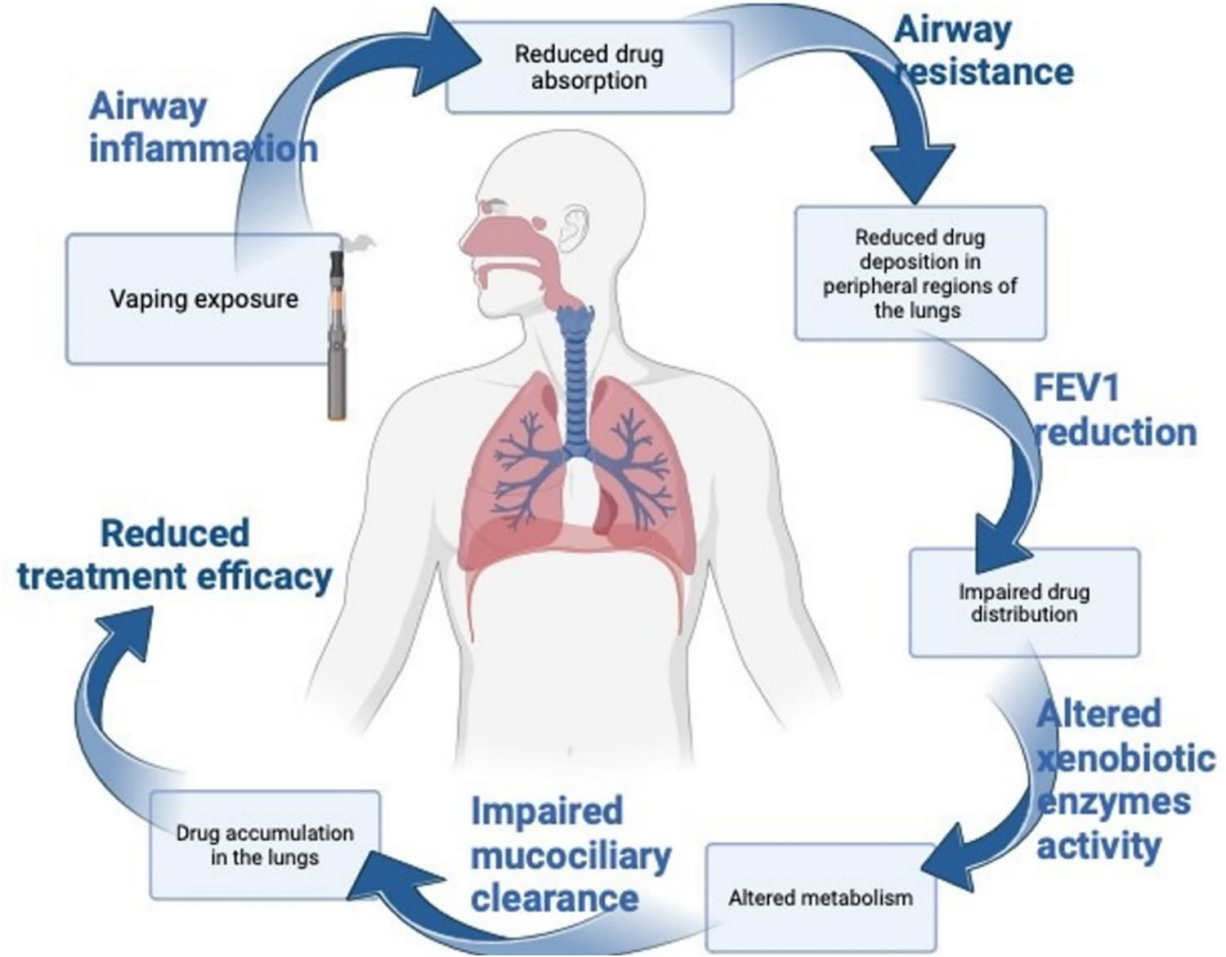
Flow diagram illustrating the impact of e-cigarette vapour on the efficacy of inhaled therapeutics, leading to decreased absorption through stepwise changes in the pulmonary anatomy (*Illustration created with BioRender)*

In summation, vapour exposure induces airway inflammation through its interactions with the NF-κB pathway, reducing drug absorption (Liu et al. 2017). Airway resistance and subsequently FEV1 reduction can lead to reduced distribution of the drug (Guo et al. 2021). Additionally, vaping directly impacts xenobiotic enzyme activity, leading to altered drug metabolism. Xenobiotics may be upregulated or inhibited in response to vapour exposure, resulting in an increase or decrease in metabolic function, respectively (Enlo-Scott et al. 2021). Consequently, drug accumulation in the lungs is altered through the impairment of mucociliary clearance, reducing overall treatment efficacy, as shown in Fig. 1.

## Conclusion and future perspectives

Vaping and e-cigarettes initially emerged as a healthier alternative for smoking, with manufacturers promoting it as a safer option than traditional cigarettes. However, evidence regarding its safety is contradictory. Whilst it is suggested that e-cigarettes may assist with smoking cessation, showing higher adherence rates and reducing withdrawal symptoms compared to nicotine replacement therapy, their consumption has also been linked to dual use, relapse and pulmonary tissue damage. The increasing prevalence of e-cigarette use, particularly among younger populations, highlights the importance of a greater understanding surrounding its health impacts. Current concerns include the risk of ongoing nicotine addiction, the introduction of harmful substances such as heavy metals and carcinogens into the lungs and the lack of data surrounding long-term usage.

Throughout our review, we have demonstrated the various damages that vaping is suspected to impose upon the pulmonary tissue, including inflammation, oxidative stress and disruption of mucociliary clearance. We have delved into damaged lung tissues and mechanisms that may interfere with the pharmacokinetics of inhaled drugs by reducing absorption and distribution, manipulating metabolism and slowing clearance. Our findings suggest that our hypothesis has been proven correct, and e-cigarettes do mirror traditional cigarettes in that they induce pulmonary inflammation, ultimately impacting the efficacy and pharmacokinetics of inhaled therapies. Therefore, patients who use e-cigarettes may experience a decrease in the efficacy of their inhaled treatments, such as bronchodilators and corticosteroids, which are commonly used for prevalent conditions such as asthma and COPD. In addition, the prolonged retention of drugs in the lungs because of impaired clearance mechanisms can reduce the intended effects of the treatment and increase the risk of side effects. The standardised management of asthma and COPD consists of the regular use of inhaled bronchodilator and corticosteroid treatment. E-cigarette use and the irreparable damage caused may additionally cause a vicious cycle where patients may experience frequent exacerbations despite maintaining adherence to their inhaled treatments due to its inhibitory effects in normal lung processes.

The suggested damage that e-cigarette use can cause, in combination with its suspected interactions with lifesaving medications, should be considered in the management of threatening conditions such as asthma and COPD. Current therapeutic guidelines for these conditions recommend smoking cessation as an integral step in disease management. The use of e-cigarette products as a smoking cessation tool in this vulnerable population group may lead to reduced efficacy of medications and worsened patient outcomes. Consumers and health professionals alike should be made aware of this relationship and use this information to guide their health decisions and management, particularly when these devices are used in conjunction with inhaled medications.

At the time of writing this review, current legislation surrounding the manufacture and provision of these products is continuously evolving. Current Australian legislation surrounding the provision of vapes and vaping products states that they may only be supplied through a pharmacy. This ensures a pharmacist is able to review a patient’s comorbidities and medications before they are obtained. A greater understanding surrounding these products and their interactions with commonly supplied medications should be considered in reviewing these policy outcomes and could inform future legislation.

The growing popularity parallels to cigarettes and links to respiratory damage and disease associated with e-cigarettes all highlight the concerns surrounding their use in current society. We have shown that individuals who have used e-cigarette products are more likely to partake in traditional cigarette usage. Furthermore, as we have uncovered that vaping mimics the effects of traditional cigarette smoking in the lungs, dual use may lead to additive damage. This increased prevalence raises concerns about the long-term effects placed on individual health as well as the health care system. Reduced absorption may necessitate higher doses, increasing the risk of adverse effects, while reduced mucociliary clearance may put patients at greater risk of acquiring respiratory infections or pneumonia. Additionally, a higher prevalence of disease may lead to increased hospitalisation rates, imposing a financial burden while also diverting resources that could be utilised for other patients.

Given that e-cigarette use is relatively new, the existing literature lacks prospective data. This casts light on potential for future research, especially considering its growing popularity and common misconception that it is a healthier alternative to traditional cigarette smoking. Our findings, in accompaniment with further research, may be used to guide future legislation, management decisions and therapeutic guidelines surrounding the use of e-cigarette devices.

The existing research and literature on the effect of vaping on inhaled therapeutics is limited, clearly highlighting a need for further investigations. Furthermore, as e-cigarette usage is still relatively new, the available data lacks longevity. We strongly suggest that further research is performed in this area due to the growing popularity and misconceptions surrounding its safety.

## Data Availability

No new data were generated or analyzed in this study. Data sharing is not applicable to this article as it is a review.
